# Mapping the Global Distribution of Trachoma: Why an Updated Atlas Is Needed

**DOI:** 10.1371/journal.pntd.0000973

**Published:** 2011-06-28

**Authors:** Jennifer L. Smith, Danny Haddad, Sarah Polack, Emma M. Harding-Esch, Pamela J. Hooper, David C. Mabey, Anthony W. Solomon, Simon Brooker

**Affiliations:** 1 London School of Hygiene and Tropical Medicine, London, United Kingdom; 2 International Trachoma Initiative, Task Force for Global Health, Atlanta, Georgia, United States of America; 3 Kenya Medical Research Institute—Wellcome Trust Research Programme, Nairobi, Kenya; Centers for Disease Control and Prevention, United States of America

Trachoma is the leading infectious cause of blindness worldwide, and responsible for the loss of an estimated 1.3 million disability-adjusted life years, mainly in sub-Saharan Africa [1]. Geographically, trachoma is a greater public health risk in dry, dusty, and hot settings, where poor, rural communities suffer a disproportionate burden of disease. Trachoma is caused by infection with the bacterium *Chlamydia trachomatis*, but the condition has a number of clinical manifestations that are the consequences of current or past infection [2]. Prevalence of infection and clinical signs of follicular conjunctivitis are highest in children under 10 y of age. Recurrent episodes of infection and associated inflammation can cause scarring, visual impairment, and potential blindness later in life.

In 1998, the World Health Organization (WHO) established an international alliance eliminate trachoma as a public health problem by 2020, called the Alliance for Global Elimination of Trachoma by the year 2020 (GET2020). The goal of GET2020 is to reduce the burden of trachoma in any community to less than one case of trachomatous trichiasis (TT) per 1,000 total population, and to less than 5% prevalence of “trachomatous inflammation–follicular” (TF) in children aged 1–9 y [3]. The strategy employed to reach these targets is based on a combination of interventions known as SAFE: Surgery to correct trichiasis, Antibiotics for *C. trachomatis* infection, Facial cleanliness, and Environmental improvement to reduce transmission. In order to allow time for programme implementation and impact by 2020, it will be necessary to scale up to full SAFE implementation in all endemic districts by 2015. An essential component of this effort is producing up-to-date and accessible maps of the distribution of trachoma for advocacy, planning, and operational research purposes.

## Why Are Updated Maps Important for Trachoma and Its Control?

No one would question the importance of a geographical understanding of trachoma in the initial planning of national control programmes. However, there are important reasons why we need to continue to update our geographical understanding of trachoma as the program is implemented, and why maps should be made publicly available. First, and the importance of this reason should not be underestimated, maps play a vital advocacy role, showcasing the achievements of control efforts to date and showing where further control resources are still required. This advocacy will help leverage continual funding.

Second, updated maps can help refine ongoing control efforts by identifying where control has been successful in reducing the burden of trachoma and where it has been less so, indicating where efforts need to be intensified in order to achieve the goals of GET2020. Numerous survey data have been collected since the implementation of national control programmes in many endemic countries [4], [5], and inclusion of these data into updated maps provides a better information base for the evaluation of disease distributions and future planning of control efforts. It is true that in some countries, the most recent information may already be available to control managers, but this may not always be the case, and both the requisite time and technical capacity to quickly update maps are often lacking. In Southern Sudan, for example, a recent mapping exercise collated all available trachoma data into a single database for the first time and provided valuable information for planning control [6]. Updated maps can help identify where epidemiological data are still absent and highlight where additional surveys are required to define future control needs. Furthermore, maps allow programme managers to more fully explore patterns of endemicity on the borders of their programme area, which may prompt further survey work and coordination of control efforts within populations that are mobile across those borders.

Third, updated trachoma maps are essential for deriving reliable estimates of the disease burden of trachoma. A recent review by Burton and Mabey [7] highlighted the unreliability of current global prevalence estimates, which is partly due to extrapolation of limited estimates across countries and regions. Collation, mapping, and analysis of data collected through cluster population sampling [3] can help generate more precise estimates of the burden of trachoma within a country, as well as allow a better evaluation of the geographical overlap with other neglected tropical diseases. This evaluation can take advantage of other ongoing efforts to map the global distribution of schistosomiasis and soil-transmitted helminth infections [8], [9].

Finally, high-resolution mapping of trachoma has a number of operational research applications, as indicated below.

## A Trachoma Mapping Project

In 2005, the International Centre for Eye Health at the London School of Hygiene and Tropical Medicine, in collaboration with the Programme for the Prevention of Blindness and Deafness at the WHO, developed a first global atlas of trachoma using data consolidated at the district level (defined as the normal administrative level for health care management) [10]. A new collaboration between the International Trachoma Initiative and the London School of Hygiene and Tropical Medicine is updating this atlas to reflect the changing burden of trachoma, while also allowing for changing administrative boundaries and providing a flexible approach in calculating district-level estimates of disease burden. The aim of the current mapping project is two-fold: (i) to provide district-level prevalence maps of the current distribution of trachoma for all implementation partners, and (ii) to collate and geolocate disaggregated estimates of trachoma by survey cluster for operational research purposes. Updated district-level maps were made publicly available in early 2011 on an open-access Web site (http://www.trachomaatlas.org/). We are continuing to collate data and update maps in real time whenever further data are received. Such an open-access information resource complements recent efforts to develop a Global Atlas of Helminth Infection (http://www.thiswormyworld.org/) [8] launched in 2010.

## What Data Should Be Included in the Trachoma Atlas?

The burden of trachoma is usually assessed using one of three survey methodologies: population-based prevalence surveys (PBPSs), acceptance sampling trachoma rapid assessment (ASTRA), or trachoma rapid assessment (TRA). While each survey methodology has its relative benefits and limitations [11], PBPSs remain the gold standard for estimating prevalence at the district level. However, ASTRA and TRA can also contribute to our understanding of the global distribution of trachoma. In particular, TRAs provide information on whether trachoma is present or absent in the most vulnerable populations within a district or community. ASTRA employs an adaptation of lot quality assurance sampling, which can give valid cluster-level prevalence estimates with varying precision and, if nested within an area-sampling framework, may provide finer spatial resolution than other methods [12]. To date, lot quality assurance sampling methodologies have been shown to provide a rapid, valid, and cost-effective approach for identifying communities requiring interventions for schistosomiasis and other neglected tropical diseases [13], and are being investigated for use in assessing the burden of trachoma. It will be important to establish to what extent trachoma data collected using these differing surveys methods can be integrated into a single cartographic platform, and this is an important research priority.

Epidemiological data on trachoma are typically based on diagnosis using ocular examination, predominantly using the 1987 WHO simplified grading system. This system was developed for the simple and reliable grading of trachoma clinical signs by non-specialist health personnel, as ophthalmologists are in short supply in the resource-poor settings in which trachoma is found [14]. Laboratory methods, such as polymerase chain reaction (PCR)–based assays, provide information on presence or absence of *C. trachomatis* infection [14], but currently remain largely confined to research studies because of their high cost. In the future, however, alternative rapid diagnostic tests for trachoma may be available for mapping trachoma.

To assemble the updated global atlas of trachoma available at http://www.trachomaatlas.org/, we used a combination of electronic literature searches, manual searches of private journal collections, and contact with a network of trachoma researchers and programme managers. Identified studies are subject to a strict review process, in which representative prevalence estimates from PBPSs and ASTRA sampling techniques are included in the database. In keeping with relevant control guidelines, the core indicators represented in the atlas are TF in children aged 1–9 y and TT in adults over the age of 14 y. Despite our focus on surveys that use standard methods and indicators recommended by the WHO, TRA surveys can be used to indicate the presence or absence of disease where PBPSs are lacking. Relevant content included in the database is summarised in Box 1. To avoid cluster-level data being used as prevalence estimates for communities, only district-level maps are available on the Web site.

**Box 1. Database Content**

Although preferred clinical indicators are TF in children aged 1–9 years and TT in adults over 14 y, alternative indicators and age groups are recorded based on availability. For each district and cluster, the following data are requested:

- Site name (district or village name) and type of location (district or village)
- Longitude and latitude of community or cluster (if available)
- Region and district in which the survey was done
- Survey methodology and population sampling
- Year of survey
- Active trachoma
  - Clinical indicator (TF, TF/TI^a^, or TI)
  - Age range
  - Number examined
  - Number graded positive
- Trichiasis
  - Age range
  - Number examined
  - Number graded positive for TT

^a^TI, trachomatous inflammation–intense.

## Why Include Cluster-Level Data in the Atlas?

Trachoma is a focal disease, and reduction in prevalence has been observed to occur unevenly throughout a district [12]. As overall transmission of *C. trachomatis* is reduced, the spatial heterogeneity of infection is likely to increase. Higher resolution mapping of trachoma will therefore help identify and target high-prevalence foci over different stages of control. Fine-scale mapping of trachoma has two important potential research applications. First, it can inform the refinement of mapping strategies, particularly in settings where trachoma reaches very low levels. The ability of alternative survey designs to accurately identify remaining disease foci can be investigated using spatial analysis and conditional simulation [15].

Second, it may be possible to generate risk maps of trachoma based on environmental and other spatial covariates. Trachoma has strong and consistent links with poverty, the effects of which may influence observed associations with climatic factors, water availability, latrine access, and standard of hygiene [16]. Other studies have reported the risk of trachoma to vary in relation to altitude, which may influence the density of flies and thus transmission through these vectors [17], [18]. Environmental and climatic data are nowadays readily available through satellite data, and integration of such data with estimates of trachoma prevalence may help predict the risk of trachoma in unsampled locations. The potential of spatial analysis of trachoma and risk mapping has recently been demonstrated for Southern Sudan [6].

## Potential Limitations

An obvious limitation of our updated atlas is the inherent variation in the quality and availability of data between included studies. Older surveys may not capture recent reductions in trachoma due to improvements in socioeconomic status, water and sanitation, and hygienic behaviours. In addition, although the WHO's simplified grading system has good inter- and intra-observer agreement, other aspects of survey design may limit the precision of reported prevalence estimates, including the number of clusters surveyed and the administrative unit for which the survey was representative. Differences in sampling methodology, the clinical indicator reported, and age groups surveyed will also reduce comparability of data; however, inclusion of this information in the database will allow stratification of data and possible calibration of data to enhance data comparability. Finally, although there has been an increase in available survey data in recent years, there remains a notable lack of information for much of Asia.

## Future Applications and Conclusion

**Figure 1:**
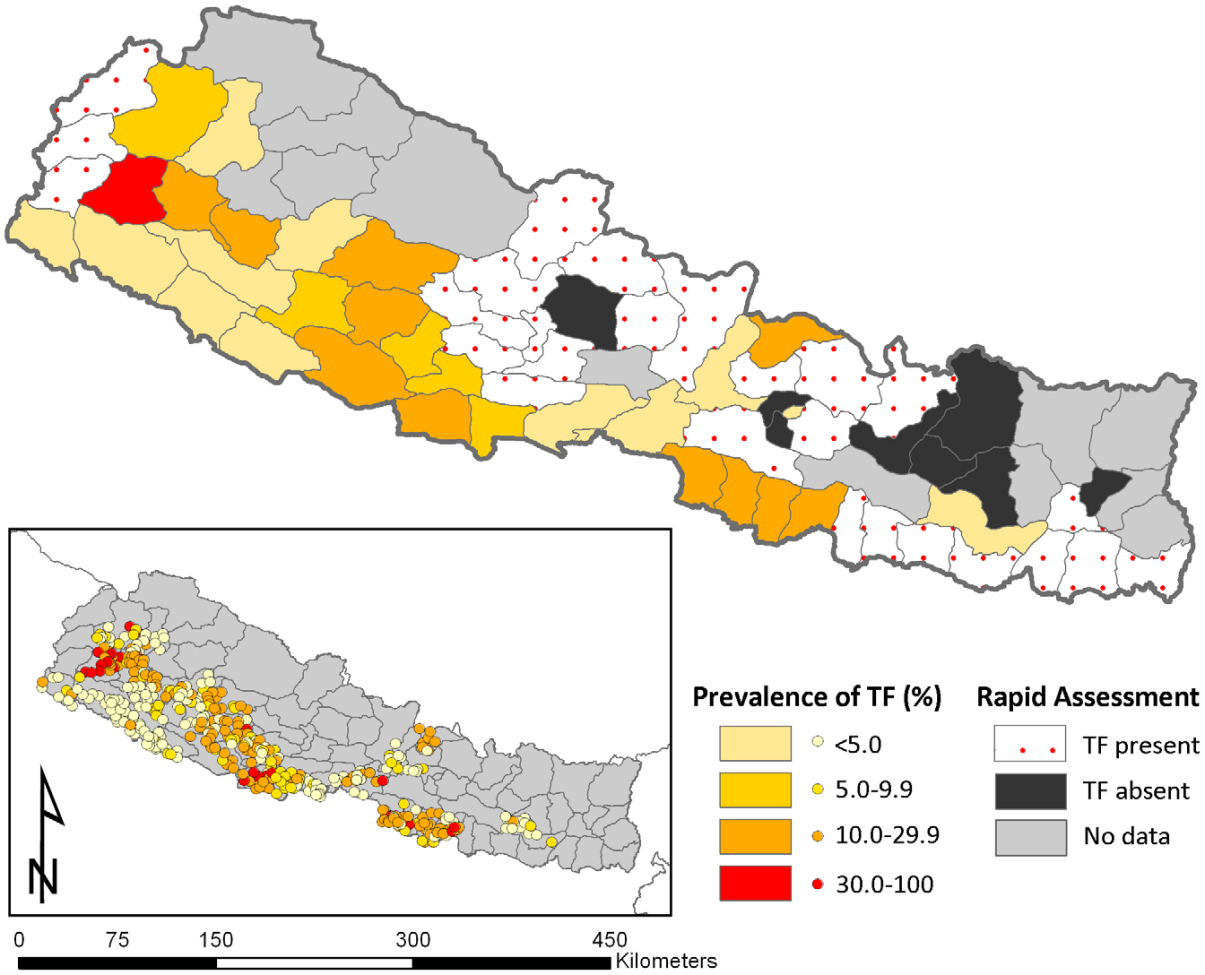
Map of Nepal showing district-level prevalence estimates of TF in children aged 1–9 years. In the updated trachoma atlas, district-level maps of each country, such as this one for Nepal, categorise prevalence according to WHO treatment thresholds, and include TRA surveys to indicate the presence or absence of trachoma. The inset shows disaggregated cluster estimates based on the most recent PBPS data. This map might be used to advocate for further support for PBPSs in areas where trachoma has been found by TRA surveys. An exploratory analysis of the spatial structure of trachoma in Nepal using cluster data shows marked heterogeneity within districts, and a clear spatial structure that could be used to generate interpolated maps and prioritize data collection in unsurveyed districts.

The new trachoma atlas, which has recently been made available on an open-access Web site (http://www.trachomaatlas.org/), provides an important resource for all stakeholders involved in trachoma control. For countries without national programmes or in need of massive scale up, the atlas will likely provide a tool to determine treatment needs, enable more effective monitoring and evaluation of ongoing control activities, and help identify control needs through a gap analysis. An example of the maps to be produced is shown in Figure 1. The current trachoma atlas initiative represents a renewed effort to update the global distribution of endemic trachoma, and to both provide maps for all implementation partners and consolidate disaggregated data for future detailed mapping and operational research. The trachoma atlas may be used alongside maps of the geographical implementation of SAFE interventions at sub-national levels, recently made available by the International Coalition for Trachoma Control (http://www.trachomacoalition.org/). It is hoped that these atlases will be a valuable resource accessible to all stakeholders, but their utility will depend on a collaborative effort by the international trachoma community to ensure that the most up-to-date data are included and that the developed maps are effectively used.
